# Modelling an optimum vaccination strategy against ZIKA virus for outbreak use

**DOI:** 10.1017/S0950268819000712

**Published:** 2019-05-16

**Authors:** Eduardo Massad, Francisco Antonio Bezerra Coutinho, Annelies Wilder-Smith

**Affiliations:** 1 School of Medicine, University of Sao Paulo and LIM01-HCFMUSP, Sao Paulo, Brazil; 2 School of Applied Mathematics, FundaçãoGetúlio Vargas, Rio de Janeiro, Brazil; 3 Germany g Department Public Health and Clinical; Heidelberg Institute of Global Health, University of Heidelberg; 4 Medicine, Epidemiology and Global Health, Umeå University, SE-901 85 Umeå, Sweden; 5 Department of Disease Control, London School of Hygiene and Tropical Medicine, UK

**Keywords:** Control strategies, mathematical models, vaccines, Zika virus

## Abstract

We present a model to optimise a vaccination campaign aiming to prevent or to curb a Zika virus outbreak. We show that the optimum vaccination strategy to reduce the number of cases by a mass vaccination campaign should start when the *Aedes* mosquitoes' density reaches the threshold of 1.5 mosquitoes per humans, the moment the reproduction number crosses one. The maximum time it is advisable to wait for the introduction of a vaccination campaign is when the first ZIKV case is identified, although this would not be as effective to minimise the number of infections as when the mosquitoes' density crosses the critical threshold. This suboptimum strategy, however, would still curb the outbreak. In both cases, the catch up strategy should aim to vaccinate at least 25% of the target population during a concentrated effort of 1 month immediately after identifying the threshold. This is the time taken to accumulate the herd immunity threshold of 56.5%. These calculations were done based on theoretical assumptions that vaccine implementation would be feasible within a very short time frame.

## Introduction

Since its introduction in the Brazilian Northeastern cities of Recife and Salvador in 2014 by travellers from French Polynesia [1], the mosquito-borne flavivirus Zika virus (ZIKV) spread rapidly and in less than a year, cases emerged throughout South America and the Caribbean, and in parts of North America. ZIKV has raised global concerns due to its association with birth defects and Guillain–Barré syndrome [2]. The magnitude of ZIKV outbreaks in the Pacific region and in particular in Brazil in 2015, led the World Health Organization to declare ZIKV a ‘Public Health Emergency of International Concern’ [3].

Zika also affected travellers [4–9]. The rapid spread of ZIKV via travellers to other American countries, where it caused an estimated 1.9 million infections [10, 11], including its potential spreading to Europe [12, 13], has spurred the search for an effective vaccine [14].

The list of candidate vaccines has reduced from 45 in 2017 to only 12 in the test, with three candidates in phase II evaluation [15]. The candidates are based on neutralizing antibodies by nucleic acid vaccines, inactivated virions and live and attenuated virus, all with the objective of stimulating neutralisation antibodies against antigens in the surface of the virion [16]. Other ZIKV vaccine developments are based on antibodies derived from dengue infection, which showed neutralisation potential against ZIV [7]. DNA vaccines are particularly interesting because they have the potential to be rapidly scaled-up and deployed during an outbreak should they prove to be efficacious. DNA vaccines work by creating a plasmid (a circle of DNA) that encodes the genes for the virus's envelope proteins. Once this plasmid makes its way into the nucleus of the target cell, the cell's own molecular machinery begins producing the viral proteins, which then induce an immune response [17].

However, the brief nature of the 2015–2016 outbreaks is making wide-scale testing of the new vaccines difficult and some research/development programmes have already been curtailed [18, 19]. Even if an effective vaccine against ZIKV manages to reach the market in the near future, many questions remain to be answered. Should a vaccine be introduced in response to an imminent outbreak or should it be introduced in the routine immunisation programme? In the case of an outbreak vaccine, when should it be introduced and for how long? In the case of a routine vaccine, which age should be targeted? For the time being, WHO has only developed a target product profile for a Zika vaccine for outbreak use [20].

We aim to address the scenario of an outbreak use of Zika vaccines. In particular, we aim to determine the optimum time point to introduce a mass vaccination campaign in response to an outbreak, the thresholds to declare an outbreak, the optimum duration of such a strategy and the coverage needed to avert an outbreak.

## Methods

The method used to answer the questions of when to trigger a ZIKV vaccine in response to an outbreak and how long should a vaccination campaign last consists of:

1. Fitting the incidence of dengue in an area relatively free of ZIKV. We have chosen the city of Rio de Janeiro in the year between October 2011 and September 2012;
2. From the fitted incidence of dengue, we calculated the total number of *Aedesa egypti* mosquitoes according to the methods detailed in [21, 22];
3. The number of *Aedes* mosquitoes in the chosen are and the period was used in a dynamical model detailed below to estimate how different vaccination schemes would minimise the number of ZIKV cases in case of an outbreak. We modelled the optimum moment to introduce the vaccine and the optimum duration of a vaccination campaign.

### Outbreak use of a vaccine with entomological indices as a trigger

We begin by fitting a continuous function, denoted Incidence_DENV_(*t*), to the number of reported ZIKV cases. The incidence data were fitted to the chosen function [21, 22]:
1
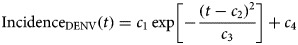

representing the time-dependent dengue infection incidence. In equation (1) *c*_1_ is a scale parameter that determines the maximum incidence, *c*_2_ is the time at which the maximum incidence is reached, *c*_3_ represents the width of the time-dependent incidence function and *c*_4_ is just another scaling parameter. Equation (1) is intended to reproduce a ‘Gaussian’ curve and so *c*_1_ and *c*_4_ are just scale parameters but *c*_2_ represents the ‘mean’ (and mode or maximum) time and *c*_3_ represents the ‘variance’ of the time distribution of cases. All parameters *c*_*i*_, *i* = 1, …, 4 were fitted to model (1) by the Bootstrap method [23] the results of which are shown in Table 1.

**Table 1.** Parameters' values (mean, lower bound and upper bound) fitted to equation (1) to ZIKV incidence in Salvador 2015 by Bootstrap technique [23]

Parameter	Mean	Lower bound	Upper bound
*c*_1_	10 982	10 075	11 937
*c*_2_	7.2362	7.1716	7.2872
*c*_3_	3.7351	2.3715	4.0633
*c*_4_	278.296	257.681	301.512

**Fig. 1.**
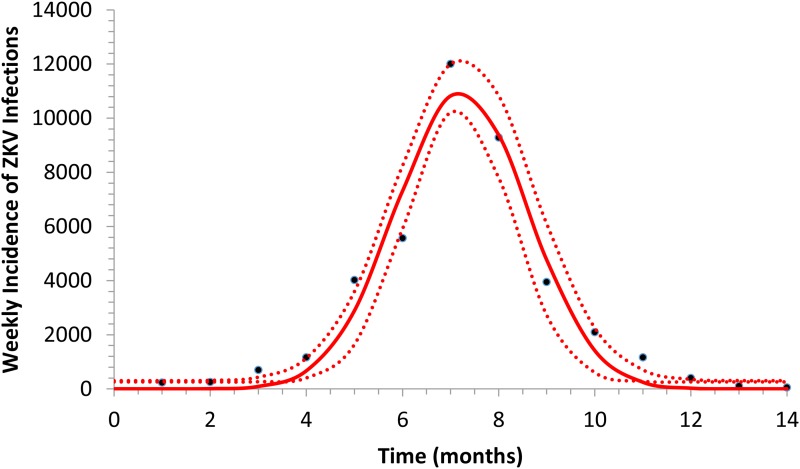
Fitting a function (eq. 1) to ZIKV incidence of infections in Salvador in 2015. Continuous line represents the mean curve whereas the finely dotted line the 95% CI.

Figure 1 illustrates the fitting of equation (1) to the incidence data of ZIKV in Salvador in 2015.

Next, we calculated the total number of *Aedes* mosquitoes in Salvador in 2015 according to the method detailed in [21, 22]. We begin by defining the incidence as the product of the force of infection, *λ*(*t*), times the number of susceptible individuals, *S*_H_(*t*). The force of infection is defined as *λ*(*t*) = (*abI*_M_(*t*))/(*N*_H_(*t*)), where *N*_H_(*t*) denotes the total human population, *a* is the mosquitoe biting rate, *b* is the fraction of those bites produced by the infectious mosquitoes *I*_M_(*t*) that are infective to susceptible humans *S*_H_(*t*). All the parameters used in these calculations and in the model simulations are shown in Table 2 and were obtained by the Latin Hypercube method [24] that best reproduced the ZIKV outbreak in Salvador in 2015.

**Table 2.** Model parameters, biological meaning and values. The dimension of rates is months^−1^

Parameter	Meaning	Mean
*a*	*A. aegypti* biting rate	25.400
*b*_dengue_	Fraction of bites actually infective to humans (dengue)	0.700
*μ*_H_	Human natural mortality rate	1.096 × 10^−3^
*γ*_H−D_	Dengue recovery rate	1.500
*γ*_M−D_	Latency rate in mosquitoes for dengue	4.000
*γ*_H−ZIKV_	ZIKV recovery rate	1.500
*γ*_M−ZIKV_	Latency rate in mosquitoes for ZIKV	4.000
*μ*_M_	Natural mortality rate of mosquitoes	2.330
*c*_dengue_	*A. aegypti* susceptibility to dengue	0.700
*b*_ZIKV_	Fraction of bites actually infective to humans (ZIKV)	0.610
*c*_ZIKV_	*A. aegypti* susceptibility to ZIKV	0.530
*r*_H_	Humans reproduction rate	0.017
*K*_H_	Humans carrying capacity	7.000 × 10^6^
*g*	Proportion of ZKV-infected mothers	0.500
*p*	Proportion of ZIKV-infected babies who develop Zika congenital syndrome	0.060
α_H_	Death rate of babies with Zika congenital syndrome	5.000 × 10^−3^
*σ*	Rate of microcephaly development	8.000

From the fitted incidence, Incidence_DENV_(*t*) we calculate the number of infective mosquitoes

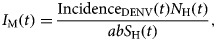

where *S*_H_(*t*) is the entire Salvador population in 2015. From the number of infectious mosquitoes, we calculated the number of latent mosquitoes, *L*_M_(*t*), given by

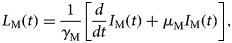

where 1/*γ*_M_ is the average duration of the extrinsic incubation period and *μ*_M_ is the natural mortality rate of mosquitoes. The number of susceptible mosquitoes is given by

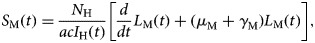

where *c* is the fraction of the mosquitoes that bite infective humans and acquire the infection, and *I*_H_(*t*) is the number of humans infected with dengue. The estimated total number of mosquitoes is *N*_M_(*t*) = *S*_M_(*t*) + *L*_M_(*t*) + *I*_M_(*t*). We are going to need the derivatives of these functions:
2
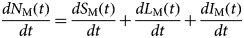


From the total mosquitoes' population curve, we calculate the mosquitoes' densities, *m*(*t*), defined as the ratio between the number of mosquitoes with respect to the human population. The mosquitoes' density is one of the components of the Effective Reproduction Number, *R*(*t*), such that:
3
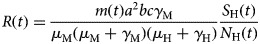


From equation (3) it is possible to estimate the critical value of the mosquitoes' density, *m*_crit_, that would guarantee that *R*_0_ > 1, that is:
4
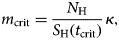

where
5
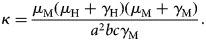


**Fig. 2.**
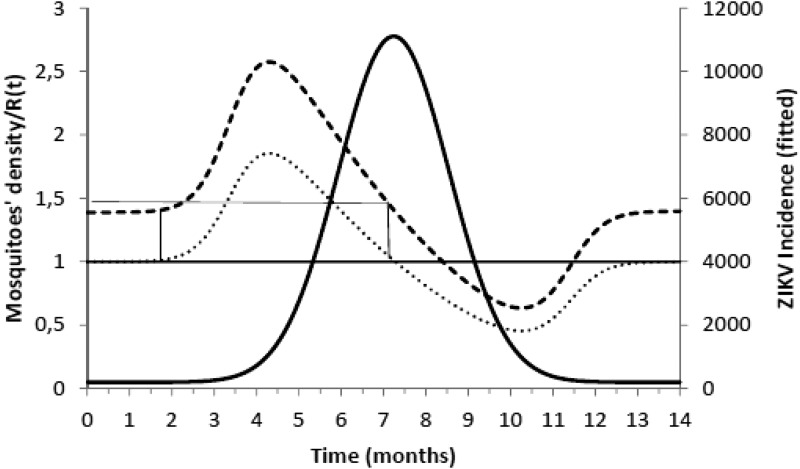
ZIKV incidence of infections in Salvador in 2015 (continuous line), Effective Reproduction Number (finely dotted line) and the mosquitoes' density (grossly dotted line). The thin line represents the threshold for transmission whereas the very thin line the critical mosquitoes' density that marks the moment *R*(*t*) crosses 1 from below and from above. The simulation used the parameters as in Table 2.

Figure 2 shows the fitted incidence of ZIKV in Salvador (2015) along with the curves of the Effective Reproduction Number, *R*(*t*), and the mosquitoes' densities *m*(*t*).

### Outbreak use of a vaccine with human cases as a trigger

It can be noted from Figure 2 that *R*(*t*) crosses the unit threshold at *m*_crit_(*t*) slightly below 1.5 mosquitoes per human. This value will be used to determine the critical moment and a preventative vaccine should be introduced. For this we have chosen the case of the city of Rio de Janeiro for the dengue season of 2011–2012, the year with the highest dengue incidence in history so far. From dengue incidence in that period, we calculated the total number of *Aedes* mosquitoes in order to determine the critical moment the mosquitoes' densities crossed the 1.5 mosquitoes per human threshold. That should be the moment when in case ZIKV-infected travellers arrival could trigger a ZIKV outbreak. It should be, therefore, the moment to introduce the preventative vaccine in the form of a universal campaign aiming both sexes and all age groups. We are assuming that the critical mosquitoes' densities for Salvador and Rio de Janeiro are the same because the basic reproduction number of Zika for both cities is quite similar in value (2.33; CI 1.97–2.97 for Rio de Janeiro [25] and 2.1; CI 1.8–2.5 for Salvador [26]).

The mosquitoes' densities for Rio de Janeiro in 2011–2012 were calculated by the same method as described above for Salvador in 2015, by fitting the dengue incidence data to equation (2) and then proceed with the calculations described above. The fitting parameters to dengue incidence are described in Table 3.

**Table 3.** Parameters' values (mean, lower bound and upper bound) fitted to equation (1) to dengue incidence in Rio de Janeiro 2011–2012 by Bootstrap technique [23]

Parameter	Mean	Lower bound	Upper bound
*c*_1_	147 144	134 944	159 879
*c*_2_	10.004	9.9147	10.073
*c*_3_	3.49035	22 158	4.0631
*c*_4_	1500.02	1388.89	1625.31

**Fig. 3.**
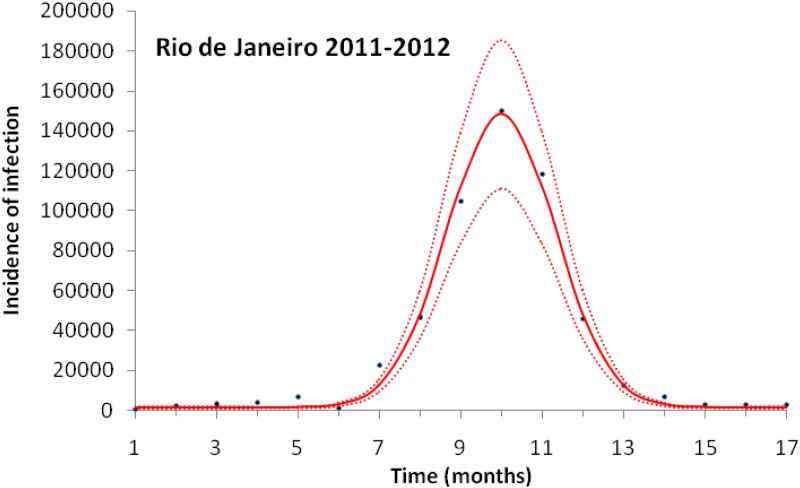
Fitting a function (eq. 1) to dengue incidence of infections between October 2011 and December 2012 in Rio de Janeiro. Dots represent the notified data multiplied by 4, continuous line the mean fitted incidence and dotted lines de 95% CI.

Figure 3 illustrates the case of 2011–2012, the years with the outbreak with the highest incidence of dengue in the history of Rio de Janeiro, Brazil.

**Fig. 4.**
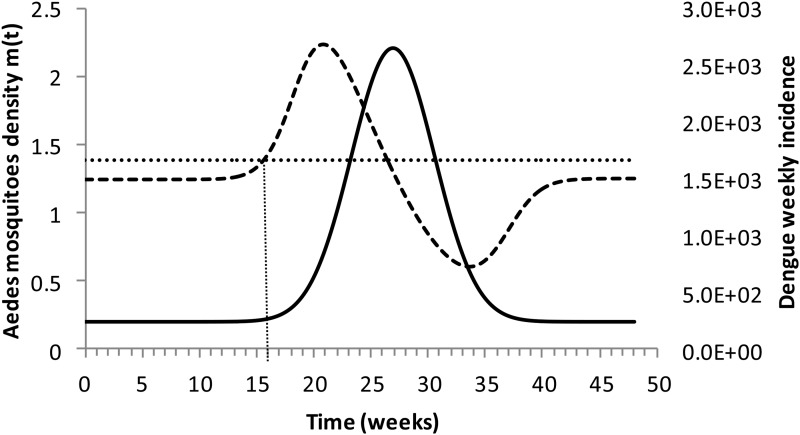
Dengue incidence of infections between October 2011 and December 2012 fitted to equation (1) (continuous line) and the mosquitoes' densities (grossly dotted line). The finely dotted line represents the critical mosquitoes' density threshold (1.5 mosquitoes per humans) and the very finely dotted line the moment the mosquitoes' density crosses the threshold.

Figure 4 shows the dengue incidence fitted to equation (1) and the mosquitoes' density calculated from the total number of mosquitoes.

It can be noted from Figure (4) that at week 16 the mosquitoes' density crosses the threshold 1.5 mosquitoes per humans. From this moment onwards, ZIKV virus could invade Rio de Janeiro and would be the optimum time to introduce a preventative vaccine.

The populations involved in the transmission are human hosts and mosquitoes. Therefore, the population densities per unit area are divided into the compartments/variables described in Table 4.

**Table 4.** Model variables and their biological meanings

Variable	Meaning
*N*_H_(*t*)	Total human population
*S*_H_(*t*)	Density of susceptible humans
*I*_H_(*t*)	Density of infected humans
*I*_H_^T^(*t*)	Density of ZIKV-infected travellers
*R*_H_(*t*)	Density of recovered humans
*V*_H_(*t*)	Density of vaccinated humans
	Density of infected babies
*R*_B_(*t*)	Density of recovered babies
*M*_H_(*t*)	Density of congenital Zika syndrome babies
*N*_M_(*t*)	Total Mosquitoes Population
*S*_M_(*t*)	Density of susceptible mosquitoes
*L*_M_(*t*)	Density of latent mosquitoes
*I*_M_(*t*)	Density of infected and infectious mosquitoes

The parameters appearing in the model are defined in Table 2.

The model assumes that one or more ZIKV-infected travellers arrive at the previously unaffected area at time *t*_*x*_ and remain infected for an average period of 1/(*μ*_H_ + *γ*_H_) months, after which they either die or recover from the infection, that is 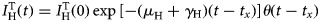. This assumption is necessary only to model Zika introduction into the system.

The model is described by the following set of equations:
6
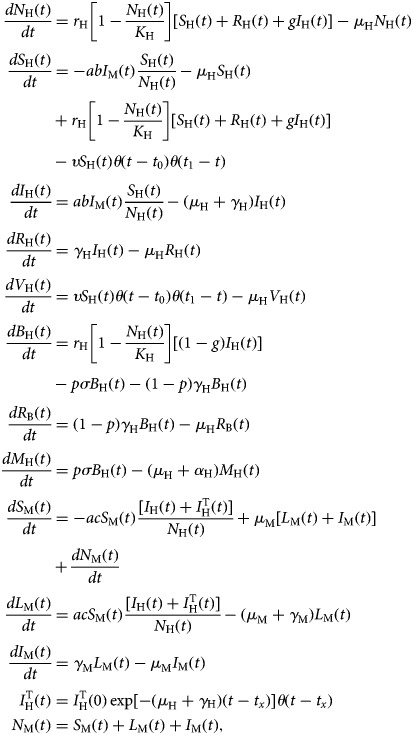

where *θ*(*t* − *t*_*x*_) is the Heaviside step function introduced to mimic the moment the vaccination starts and the moment an infected traveller arrives in the area (*t*_*x*_). The latter varied along the model simulations.

For the initial conditions, we assumed that before Zika was introduced in Rio de Janeiro, the population was at steady state, such that *S*_H_(0) = *K*_H_((*r*_H_ − *μ*_H_)/*r*_H_). The other initial values assumed were *I*_H_^T^(0) = 10, and all the remaining values were set equal to zero. The human carrying capacity was set as equal to the total population of Rio de Janeiro, that is *K*_H_ = 6.32 × 10^6^. We are well aware that the time period where the strategies are designed is very short where one would not expect density-dependent demographic effects to be important. Notwithstanding, we considered a logistic grow for the human populations for the sake of completeness of the model.

Let us explain in detail the dynamics of each described by system (6).

The total human population, *N*_H_(*t*), grows by births, expressed in the positive term of the first equation of system (6),

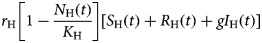

and decreases by natural deaths (i.e. deaths by other causes not related to ZIKV infection), the negative term *μ*_H_*N*_H_(*t*). Note that all susceptible and recovered individuals reproduce but only a fraction *g* of the infected contributes to the next generation of humans.

Subpopulation of susceptible humans, *S*_H_(*t*), acquire the infection from infected mosquitoes with the incidence expressed in the first negative term of the second equation of system (6), *abI*_M_(*t*) (*S*_H_(*t*))/(*N*_H_(*t*)), increases by births, expressed in the positive term,

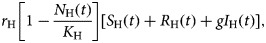

and is reduced by natural deaths, the second negative term, *μ*_H_*S*_H_(*t*), or vaccination, the third negative term, υ *S*_H_(*t*)*θ*(*t* − *t*_0_)*θ*(*t*_1_ − *t*). The two *θ* functions, *θ*(*t* − *t*_0_)*θ*(*t*_1_ − *t*), were included to simulate a square pulse that begins at *t*_0_ and last for (*t*_1_ − *t*_0_) time units.

The infected humans' compartment *I*_H_(*t*) grows by the incidence of the infection, the positive term of the third equation in system (6), *abI*_M_(*t*) (*S*_H_(*t*))/(*N*_H_(*t*)), and is reduced by deaths or recovery from infection, the negative term (*μ*_H_ + *γ*_H_)*I*_H_(*t*).

Recovered humans, *R*_H_(*t*), come from the infected with the recovery rate *γ*_H_, the positive term in the fourth equation of system (6) and die by natural causes with the rate *μ*_H_.

Vaccinated humans, *V*_H_(*t*), come from the susceptible state with vaccination rate υ, the positive term of the fifth equation of system (6) and may die by natural causes with the rate *μ*_H_.

Infected babies, *B*_H_(*t*), are born out of the fraction (1 − *g*) of infected mothers, the positive term in the sixth equation of system (6),

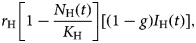

a fraction *p* of whom develops the Congenital Zika Syndrome (CZS) with rate *σ*, the first negative term and its complement (1−*p*) recover from infection with rate *γ*_H_, the second negative term.

The compartment of recovered babies, *R*_B_(*t*), grows with the positive term (1 − *p*)*γ*_H_*B*_H_(*t*), and decreases by natural deaths with the negative term *μ*_H_*B*_H_(*t*).

The last human compartment, babies with CZS, *M*_H_(*t*), grows with the positive term *pσB*_H_(*t*), and is reduced by deaths, either by natural causes with the rate *μ*_H_, or by the infection and its consequences, with the rate *α*_H_.

Susceptible mosquitoes, *S*_M_(*t*), can acquire the infection at the beginning of the outbreak from the infected travellers, *I*_H_^T^(*t*), and afterwards the disease introduction, from the autochthonous cases, *I*_H_(*t*), with the incidence represented by the first negative term of the ninth equation of system,

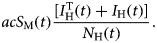


The first positive term represents the births of mosquitoes, *μ*_M_[*L*_M_(*t*) + *I*_M_(*t*)], and the second positive one by the derivative of the total mosquitoes' population, (*dN*_M_(*t*))/*dt*, calculated from dengue incidence data as explained above.

Once infected, mosquitoes pass to a latent state, described by the tenth equation of system (6), whose positive term is the incidence of the infection to mosquitoes,

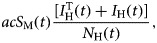

and the negative term composed by a rate of development of infectiousness (the inverse of the average extrinsic incubation period) *γ*_M_, and the natural deaths of mosquitoes, *μ*_M_.

Finally, the 11th equation of system (6) describes the dynamics of infected and infectious mosquitoes, *I*_M_(*t*), which grows by the evolution of the infectiousness state, *γ*_M_*L*_M_(*t*), and diminishes by natural deaths of mosquitoes, *μ*_M_*I*_M_(*t*).

The total number of ZIKV cases, ZIKV_cases_, is given by:
7
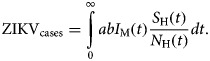


The total number of cases is given by the integral of the incidence. This does not include recovery or mortality because we are not integrating the prevalence but rather the incidence. After a given period, the number of cases is the sum of the number of new cases per time unit (incidence).

We aim to maximise vaccination effectiveness, denoted Eff, which is given by:
8
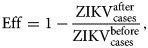

that is, 1 minus the ratio between the number of cases after vaccination with respect to the number of cases before vaccination.

## Results

**Fig. 5.**
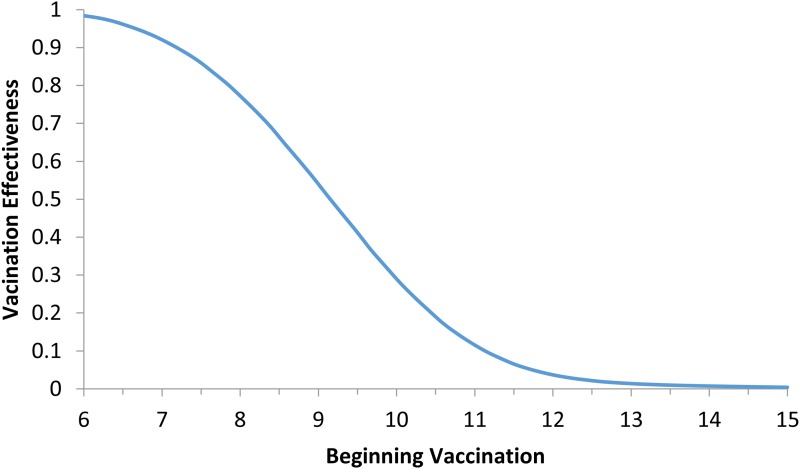
Vaccination effectiveness as a function of the moment (*t*_0_) the campaign starts.

**Fig. 6.**
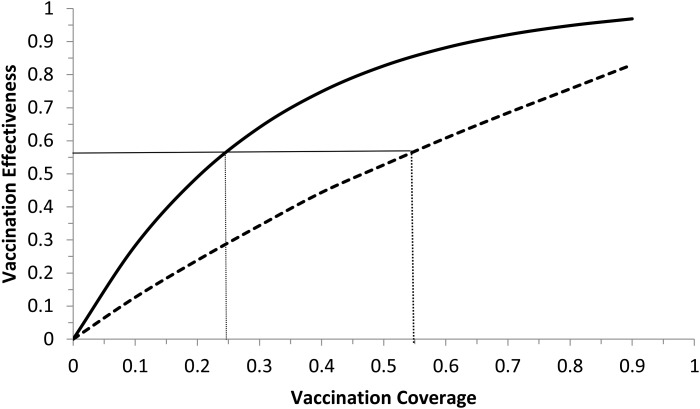
Vaccination effectiveness as a function of vaccination coverage. Continuous line represents 1 month of duration and dotted line represent 6 months of duration. The continuous horizontal line marks the herd immunity threshold from *R*_0_ ≅ 2.3.

Model (6) was numerically simulated with the parameters' values as in Table 2 in order to obtain the moment that the vaccination campaign should start (*t*_0_ in the second equation of system (6)), and the duration of the campaign (*t*_1_ − *t*_0_ in the second equation of system (6)), in both cases the aim is to maximise the vaccination effectiveness. The results are shown in Figures 5 and 6. In Figure 6, vaccination coverage Coverage was simulated from the vaccination rate υ according to:
9
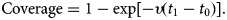

It can be noted from Figure 5 that vaccination effectiveness is maximised by starting the campaign as soon as possible. If the ZIKV-infected traveller arrives at *t*_*x*_ = 6 months (the moment that maximises the risk of ZIKV introduction in this area), the first ZIKV autochthonous case will appear 12 days later. This suggests that the vaccine should start as soon as the first ZIKV case is reported.

It can be noted from Figure 6 that vaccination effectiveness is maximised by the shorter duration (1 month). In addition, the herd immunity threshold (based on a *R*_0_ ≅ 2.3, as estimated by [25]) would be reached with a lower vaccination coverage for the 1 month campaign (~24%) than with the 6 months campaign (~54%).

Therefore, the optimum vaccination strategy would be to start a vaccination campaign at the moment the first ZIKV case is reported and concentrate the campaign with a coverage above 25% during 1 month.

## Discussion

In this work, we used the data related to the 2015 Zika outbreak in Salvador to calculate the critical mosquitoes' density that would make the basic reproduction number greater than one and so allows the infection to invade a given susceptible population. We then modelled what would be the best moment to introduce a vaccine in order to avoid the invasion of Zika infection. For this we used data from the 2011–2012 dengue season in Rio de Janeiro to check the model's predictive capacity to determine the moment the mosquitoes' density would cross the critical value. The critical mosquitoes' densities for both cities were considered the same because the basic reproduction numbers of those cities are very similar to each other.

We showed that the optimum vaccination strategy to reduce the number of cases by a mass vaccination campaign should start when the *Aedes* mosquitoes' density reaches the threshold of 1.5 mosquitoes per human, which means the time point when the reproduction number goes above one. The maximum time it is advisable to wait for the introduction of the vaccination campaign is at most when the first ZIKV case (either autochthonous or imported) is identified, although this would not be as effective to minimise the number of infections as when the mosquitoes' density crosses the critical threshold above. In both cases, however, the catch up strategy should aim to vaccinate at least 25% of the target population during a concentrated effort of 1 month. This is the time taken to accumulate the herd immunity threshold of 56.5%.

It is important to emphasise that the model presented in this paper is intended to be a method and the results of our simulations are the only illustration of a plausible scenario. These should not be taken as true public health policies but rather as an algorithm to calculate the theoretical optimum vaccination strategy. This is a modelling approach, and we are aware that determining mosquito population densities is programmatically not feasible, or very difficult to attain, on a routine basis.

We are also well aware that public health authorities do not rely on any control strategy of *Aedes*-borne infections that is based on vector control variables [27, 28]. Many years of attempt to control *Aedes* mosquitoes by adulticides, larvicides, by search-and-destroy breeding places have failed to reach any significant impact on those diseases [29]. Moreover, none of the surveillance methods used to monitor the risk of *Aedes*-borne infections (*House index*, *Container index*, *Bretau index*, *Pupa index*, *adult population density using ovitraps*, *sticky traps*, *human landing collections or any similar traps*) correlate with the number of cases [30]. However, the methods proposed in [31] to estimate the density of mosquitoes from dengue incidence data, assuming that certain parameters related to transmission are known with reasonable accuracy, provide a good estimation of the critical threshold that determines when *R*(*t*) crosses the unit threshold at *m*_crit_(*t*), as shown in Figure 4. As shown in Figure 2, *m*_crit_(*t*) in Salvador, this was reached 1 month before the number of cases of ZIKV infections started to mount up. This gives enough time to prepare and implement a mass vaccination campaign to curb the outbreak. On the other hand, if the mosquitoes' density threshold is not adopted by any reason as a criterion to trigger a vaccination campaign, then vaccination should start at most at the notification of the first case, although at this time many cases would already be occurring, given the high number of asymptomatic infections in Zika [31]. Once a vaccination campaign is initiated, the optimum strategy consists, according to our model, in continuing vaccinating the target population in a concentrated effort to reach a minimum of 25% coverage in the period of 1 month. This, as shown in Figure 3, would be enough to reach the herd immunity threshold. However, as in real life, the implementation of a vaccine strategy after notification of one single Zika case would take longer, the coverage rate would need to be higher and the effectiveness of the strategy less than optimum.

It is possible that public health authorities decide for a target vaccination (e.g. women of reproductive age only), rather than the universal campaign aiming both sexes and all age groups. Hence, for instance, vaccinating women of reproductive ages could be a possibility not considered here. However, any modification from the universal vaccination campaign simulated in this work could be easily incorporated in the model. The novelty of our approach is on determining the optimum moment to start the vaccination strategy and the duration of such an intervention rather than the nature of the strategy.

Finally, we would like to stress the fact that, even in the absence of a safe and efficacious vaccine, the theoretical model presented can help the designing of an optimum strategy to control ZIKV outbreaks the moment a future vaccine is ready to be used. Our model proposes an interesting surveillance method in which, from the incidence of dengue, an endemic infection in regions that suffered ZIKV outbreaks, one can determine the exact moment the mosquitoes' density crosses the threshold of imminent risk of an epidemic.
